# The Views of a Provincial Hospital Secretary

**Published:** 1920-06-12

**Authors:** 


### HOSPITAL FINANCE.

### The Views of a Provincial Hospital Secretary.

The problem of the hospitals is very much that of the individual with a fixed income who finds the cost of everything by which he lives increased 100 per cent. or more; but to the hospitals it is more acute and momentous. The individual reduces his standard of living, cuts off small extravagances, and suffers inconveniences, but carries on without his hard lot causing appreciable damage to the community. The hospital is in a very different position. If it contracts its functions to conform to its depreciated means it deprives the community of services which are absolutely essential. A survey of the 1919 reports shows that unless vastly increased financial support be forthcoming the hospitals will be unable to tender the services the community needs. It seems, therefore, that

(1) Hospitals must either go to the State or municipality for support.

(2) The voluntary system must receive more enthusiasm than in the past.

(3) Payment must be taken from patients.

(4) Expenditure must be reduced.

### State or Municipal Control.

At the present time the fundamental question of voluntaryism in the matter of hospitals is claiming the consideration of all thoughtful reformers. The report of the Royal Commission upon the Poor Laws states that the voluntary hospitals are doing a great work which, if they did not exist, would have to be performed by some other authorities charged with the provision of hospital accommodation. The Insurance Act, tuberculosis treatment, and later the venereal diseases treatment, military patients, and the treatment of discharged and disabled sailors and soldiers, are, by recent legislative enactments, wholly or partly a charge against public funds.

State or municipal support has advocates who maintain that as the health of the community is a national concern, so the maintenance of the hospital should be national; but they do not appear in the least concerned that the nationalisation of hospitals under a Government Department would rise to prodigious figures. Probably the only way is to have each community sufficiently interested in the support of its own hospital.

### Increased Support.

Before the hospitals make overtures for municipal or State aid they should make certain that every source of revenue has been tapped. Personally, I am of the opinion that we ourselves are to blame that our annual income does not more nearly reach the expenditure.

The advertising of hospitals' needs has in the past been upon too stereotyped a basis. Little originality and ingenuity have been displayed, and it is only latterly that there has been evidence of a desire to remedy this. What is advertising? It is simply telling about the work. We want money, so we tell the 90 per cent. who are not subscribers all about our needs; but we have to get to the 90 per cent. by means not hitherto employed. We must make our appeals telling ones. I do not mean that we must exploit the sufferings of the patients, but there is unlimited material at our hands for attractive advertisements which will hold the attention of the public and will appeal to their hearts. The appointment of a Publicity Committee for the purpose of keeping the institution's name continually before the public is worthy of consideration.

### Workpeople's Committees.

Undoubtedly a large increase of income could reasonably be looked for from those who derive advantages from the hospitals. If the Workpeople's Committees are made to realise the dire predicament in which the voluntary hospitals are placed, and are fully impressed with the urgent necessity of our needs, their sympathy will be awakened sufficiently to induce them to contribute largely towards the maintenance of the hospitals.

In my own hospital I am inviting every week parties from Workpeople's Committees to give me the opportunity of taking them over the hospital in order that they may see for themselves the work we are doing for the sick children of Birmingham and the district, and I have been encouraged by considerably increased support. They may not read an appeal, but they will come to see the hospital buildings if invited to do so, and the opportunity can be taken for telling them the hospital's wants. In showing these parties over the hospital we have to remember there are other departments than the wards and kitchens. Let them see the boiler-house, the laundry, the *x*-ray department, the electro-therapeutic department, and the clinical and pathological laboratories. Let them know that upon chemical and bacteriological research modern medicine is built, and that we cannot give proper treatment or have cases investigated properly unless there is a good chemical and bacteriological and pathological laboratory.

Let the workpeople see for themselves the good work being done for the community, and we shall not lack all the money we need. Let them realise that the hospitals are the teaching schools for the doctors and nurses, and that everyone benefits indirectly from such institutions. I have instituted the practice of taking the name of the parent's employer for both the in- and out-patients on the card-index system. At given periods a letter is sent stating the work which has been done for their employees' children, and the cost of such treatment. These letters are followed up by a call from the collector.

Representation on the Board of Governors (meeting quarterly) is given to employers, employees, and societies contributing £10 10s. a year and upwards, with a maximum of five from one firm or society for each £10 10s. subscribed. The advantages of the system are many.

### The Habit of Giving.

Let us try to cultivate into all concerned the habit of giving. We are doing it in Birmingham with the School Children of the Public Schools and the Private Schools, and we believe the interest of the children will be maintained when they arrive at man's estate. Money is now more widely distributed, and the present holders have not acquired the habit of giving, nor realised the responsibilities of their newly found wealth.

### Red Cross Fund Assistance to Voluntary Hospitals.

The scheme put forward by Sir Arthur Stanley, by which the Joint Council of the British Red Cross Society and the Order of St. John is prepared to utilise the machinery of those two bodies in the collection of funds for the provincial hospitals is of profound interest, and coming from one so intimately acquainted with the difficulties which confront the voluntary hospitals, and who

**Hospital Finance**—(*continued*).

has such first-hand experience of the Red Cross Society, the practicability of the scheme is unquestionable.

The organisation of this large band of workers who, from the experience gained during the war, have become convinced of the splendid efficiency of the British hospital system, will undoubtedly call the attention of the whole country to the present needs of the hospitals.

The provincial hospitals have in the past cast longing eyes at the King Edward's Hospital Fund and the League of Mercy. We have no such central fund capable of placing the hospitals on a sound financial basis; if the scheme of the Red Cross be accepted, the provincial hospitals will then enjoy the benefit of a great collecting organisation.

Paying Wards.

I am fully in agreement with the idea that there should be paying wards in connection with our voluntary hospitals. Many people use the hospitals without contributing to them at all, and the very few who make donations do not give anything like the proportion of their indebtedness. Let us make up our minds that we must accept the principle of taking payment from patients. It answers admirably elsewhere. There is not a general hospital in Canada or in the United States which does not take all the money it can get from all classes of patients. In New Zealand every patient must pay for his or her board.

The hospitals of the future will undoubtedly have a big private pavilion, and there is no reason to doubt that they can be made to pay. In Birmingham a private pay hospital is making tremendous strides, and in the few years it has been opened has paid its way.

Expenditure.

In considering the question of present financial difficulties, the control of expenditure presents an aspect of the subject of equal importance to that of raising funds.

I understand that some hospitals indiscriminately still give away surgical appliances at the cost of several hundred pounds a year, without making the least effort to obtain some proportion of the expense from the patients.

It seems to me there is a great need for reform in our present system of accounting. Taking the total money spent under the various headings—provisions, surgery, and dispensary, domestic, etc.—and dividing by the average number of occupied beds, does not give any credit to the economical departments, nor any guide to the extravagant ones. Cost by itself is an unreliable guide as to what a hospital is doing; but, accompanied by quantity, a true basis for estimating a hospital will be arrived at. This classification of costs under a proper system of cost accounts, by which every ward and department is separately costed, will undoubtedly bring the expenditure down very considerably.

At present the whole hospital is lumped into the common pond of cost. Individual careful users get no encouragement, and there is no incentive to be careful. Put comparative lists up each month, and the highest ward one month takes care it is not the highest the next. It saves grumbling and spasmodic efforts. The silent pressure of a comparative return works out its own salvation. Let us regulate the consumption in each department, and it will automatically regulate the cost.

